# A Patient with Slowly Progressive Proptosis

**DOI:** 10.5334/jbr-btr.1324

**Published:** 2017-09-01

**Authors:** Sven Dekeyzer

**Affiliations:** 1 OLV-Ziekenhuis Aalst, BE

**Keywords:** meningioma, sfeno-orbital meningioma, proptosis, hyperostosis

A 43-year-old woman was referred from her general practitioner to the department of radiology because of a progressive painless right-sided proptosis that had developed slowly over at least several months. There was no diplopia and thyroid function was normal. A non-contrast-enhanced Computed tomography (CT) of the brain was performed which showed an aggressive appearing sclerotic bony expansion of the right greater and lesser sphenoid wing (Figure 1). The bony lesion was associated with secondary narrowing of the right orbital compartment and proptosis. Contrast-enhanced MRI confirmed the earlier seen bony changes which were associated with contrast-enhancing thickening of the right anterotemporal dura (Figure 2). Based on the typical CT and MR characteristics, a diagnosis of spheno-orbital meningioma was proposed. Patient underwent multiple surgeries and eventually a total resection of the right lateral orbital wall and the intracranial dural component of the lesion was performed. Anatomopathology confirmed the diagnosis of a spheno-orbital meningioma.

**Figure 1:**
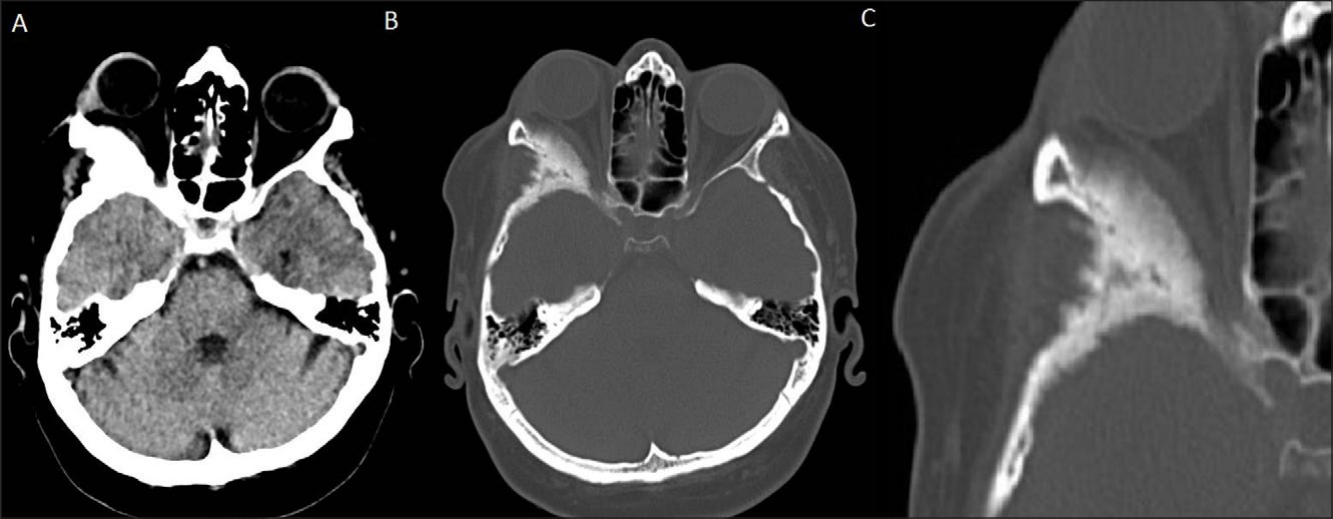
Non-contrast enhanced axial CT of the brain with soft tissue **(A)** and bony window reconstructions **(B)** shows an expansile sclerotic lesion in the right sphenoid wing. Involvement of the right lateral orbital wall leads to narrowing of the orbital space and secondary proptosis. A zoomed-in view in bony window **(C)** shows the irregularly marginated sclerotic expansion of the right lateral orbital wall in more detail.

**Figure 2:**
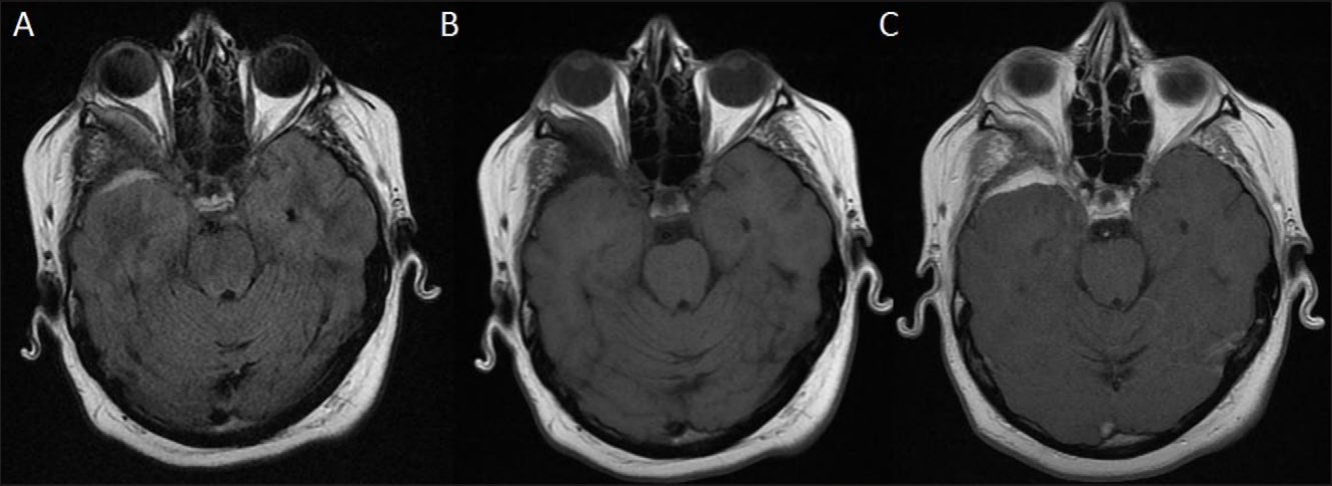
MRI of the brain with axial FLAIR **(A)** and unenhanced and contrast-enhanced axial T1-weighted images **(B, C)** shows right-sided anterotemporal FLAIR-hyperintense contrast-enhancing dural thickening abutting the bony sphenoid changes.

## Discussion

Meningiomas arise from the arachnoid cap cells of arachnoid villi and are the second most common primary brain tumors after gliomatous tumors. Sphenoid wing meningiomas are a specific subtype of meningiomas arising from the dura overlying the sphenoid wing bone. They are also known as *orbitosphenoid meningiomas* and represent about 15–20% of all meningiomas [1]. Two different growing patterns of sphenoid wing meningioma have been described: meningioma en masse, forming a nodular space-occupying lesion, and meningioma en plaque, which is flat.

Sphenoid wing meningiomas may be associated with very extensive hyperostosis, as was the case in our patient. The most accepted cause of hyperostosis associated with sphenoid wing meningiomas is direct tumor invasion, reactive bony changes, or a combination of the both. In our own patient, anatomopathology revealed islands of meningioma cells in the trabecular bone of the surgically removed right lateral orbital wall.

The most common signs and symptoms associated with sphenoid wing meningiomas are visual deterioration, proptosis and cosmetic deformity. Treatment consists of surgery and can be complex, depending on the size and extension of the tumor, with different chances of complete removal and often requiring both extensive intra- and extradural surgery. In our own patient partial resection did not lead to an improvement of clinical symptoms and in the end total resection of the right orbital wall had to be performed.
